# GIW and InCoB are advancing bioinformatics in the Asia-Pacific

**DOI:** 10.1186/1471-2105-16-S18-I1

**Published:** 2015-12-18

**Authors:** Christian Schönbach, Paul Horton, Siu-Ming Yiu, Tin Wee Tan, Shoba Ranganathan

**Affiliations:** 1 Department of Biology, School of Science and Technology, Nazarbayev University, Astana, 010000 Republic of Kazakhstan; 2 Center for AIDS Research and International Research Center for Medical Sciences, Kumamoto University, Kumamoto, 860-0811 Japan; 3 Computational Biology Research Center, National Institute of Advanced Industrial Science and Technology, Tokyo, 135-0064 Japan; 4 Department of Computational Biology, Graduate School of Frontier Sciences, University of Tokyo, Japan; 5 Department of Computer Science, Faculty of Engineering, The University of Hong Kong, Hong Kong, HKSAR; 6 Department of Biochemistry, Yong Loo Lin School of Medicine, National University of Singapore, Singapore, 117599; 7 Department of Chemistry and Biomolecular Sciences, Macquarie University, Sydney, NSW 2109 Australia

**Keywords:** Vinclozolin, Unsupervised Feature Extraction, Temporal Gene Expression Profile, Maximal Dependence Decomposition, Neddylation Site

## Abstract

GIW/InCoB2015 the joint 26th International Conference on Genome Informatics (GIW) and 14th International Conference on Bioinformatics (InCoB) held in Tokyo, September 9-11, 2015 was attended by over 200 delegates. Fifty-one out of 89 oral presentations were based on research articles accepted for publication in four BMC journal supplements and three other journals. Sixteen articles in this supplement and six articles in the BMC Systems Biology GIW/InCoB2015 Supplement are covered by this introduction. The topics range from genome informatics, protein structure informatics, image analysis to biological networks and biomarker discovery.

## Introduction

The International Conference on Bioinformatics (InCoB) is annual conference of the Asia-Pacific Bioinformatics Network [1], while GIW is the annual conference of the Association of Asian Societies for Bioinformatics (AASBi) [2]. Both GIW and InCoB are intimately associated with the development, growth and maturation of bioinformatics in Asia. Twenty-six years after the first GIW and fourteen years after the first InCoB, bioinformatics is now a well-established and diverse discipline within life and computer sciences. Hence, the differences in thrust and focus areas of the two conferences (e.g. genome informatics for GIW) faded over time. In the past five years, GIW and InCoB had to cope with increasingly tight travel budgets of researchers who are subject to stringent key performance indicators often requiring their work presented in talks to be published as articles in journals with sufficiently high impact factors. As both conferences draw submissions from a largely overlapping clientele, APBioNet and AASBi member society Japanese Society for Bioinformatics (JSBi) decided to organize a joint GIW/InCoB2015 conference [3] rather than competing for submissions and potential delegates. The immediate effects of joining forces were the ability to offer manuscript submission to seven journal tracks (Bioinformatics [4], BMC Genomics [5], BMC Bioinformatics [6], BCM Medical Genomics [7], BMC Systems Biology [8], IEEE/ACM Transactions on Computational Biology and Bioinformatics [9] and Journal of Computational Biology and Bioinformatics [10]) and savings in logistics, operational and administrative costs that allowed us to sustain moderate registration fees.

As bioinformaticians we are familiar with network topologies. If we treat conference stakeholders similar to vertices and their connections in dynamic networks and try to optimize them we can achieve a state of scale-free conferences. APBioNet considered GIW/InCoB2015 a test run for a future, potentially larger, joint multi-partner supraregional bioinformatics conference in Asia. The expected economy of scale and richness in diversity of topics will be beneficial for all stakeholders in the conference: participants, invited speakers, organizers/hosts, publishers, sponsors, funders, exhibitors, venues, transport and accommodations.

## Manuscript submission and review

All submitted manuscripts were reviewed by at least two reviewers before a decision on rejection, major revision or minor revision was reached. Details on the reviewing process, acceptance, the list of manuscripts selected for the Best Paper Awards and the names of the program committee members are available in the Introduction of the BMC Genomics GIW/InCoB2015 supplement [11]. An overview of the 16 articles in this supplement [6] and six articles in BMC Systems Biology supplement [8] is given in the next sections.

## Biomarkers and disease networks

The wider application of biomarkers in diagnosing complex diseases and their progression is still limited by suboptimal accuracy and stability. Two studies addressing these issues apply network and algorithm approaches to cardioembolic stroke and Alzheimer's disease data. Wong *et al*. [12] constructed protein-protein interaction networks based on temporal gene expression profiles derived from microarray time course data of cardioembolic stroke. Pathway analyses combined with a stroke-relevant scoring function revealed network-based biomarkers that were common or distinct for three post-stroke time points. Vandewater *et al*. [13] tackled the problem of combinatorial solution space in finding robust blood-based biomarker and demographic feature combinations that are predictive for the progression of cognitive impairment towards Alzheimer's disease. An adaptive genetic algorithm with logistic regression approach resulted in a 30 feature model that showed superior performance in predicting progression to both mild cognitive impairment and Alzheimer's disease.

Another common challenge in biomarker discovery for complex diseases is that high-throughput transcriptome, proteome and metabolome data are amenable for sub-typing (e.g. cancer) and sub-processes in the disease but require more than Gene Ontology-based functional enrichment to understand the underlying molecular mechanisms. Wu *et al*. [14] designed a large-scale text-mining system for thyroid cancer sub-classification and detailed molecular pathway understanding which appears to be sufficiently generic to be applied to other complex diseases.

Copy number alterations (CNAs) are known to contribute to the deregulation of gene expression in cancers [15]. Piccetti *et al*. [16] developed a methodology related to learning cooperative regulation networks from gene expression data [17] to derive for each gene and sample of bladder cancer with CNAs a network-wide deregulation score. The approach is expected to provide useful information on shared and different deregulation types among cancer subtypes as well as individual patients. Taguchi [18] applied principal component analysis with unsupervised feature extraction to identify deregulated genes and aberrantly methylated promoters in the endocrine inhibitor vinclozolin exposed rat F3 cell lineages representing different developmental stages. His approach yielded candidate chemokine signaling pathways and several leucine-rich repeat proteins that may play a role in transgenerational-mediated epigenetic diseases.

## Transcriptional regulation and protein-protein interactions

Multicellular human and unicellular yeast share core eukaryotic pathways that can be exploited in humanized yeast cell [19] disease models and study of transcriptional regulation. Wu *et al*. [20] implemented a one-stop web-based yeast-associated genes mining tool YAGM for biological associations that include TF binding, regulation, mutant phenotype, physical and genetic interactions among others.

Functional redundancy of TFs in yeast has been studied experimentally using knock-out strains indicating that only a small percentage of binding sites regulated by a particular TF are affected when the TF is knocked-out. Wu *et al*. [21] reanalyzed the functional redundancy for molecular features that may explain why one TF can compensate for another one. Features that rendered a TF redundant included low expression levels, TF binding sites in close vicinity of transcription start sites, few bound TFs among other factors. Benchmarking of new or modified computational methods of TF binding predictions can become time consuming when done for all methods. Lai *et al*. [22] developed a cooperative TF pair evaluator that incorporated fourteen different methods and their performance indices.

Protein-protein interactions (PPIs) can change spatially and temporally depending on the context. When PPIs are clustered, the temporal and/or spatial differences that delineate molecular biological information can be lost. Stoney *et al*. [23] designed a clustering strategy that can accommodate context dependent PPIs utilizing Gene Ontology annotations or sequence homology to determine functional communalities. The approach allows PPIs to be represented in multiple pathways rather than one node with multiple connections, a prerequisite for exploring functional organization and changes.

The work of Konishi [24] also addresses clustering in the presence of a temporal dimension. The scaled principal component analysis of mammary gland development microarray time course data showed robustness to noise and separation of groups across all time points.

## Protein structures and post-translational modifications

Post-translational modification (PTM) or the absence of it affects the structure of a protein. Potential PTM-mediated changes in protein-protein binding characteristics may alter the regulation of protein networks but also make PTM a drugable target in diseases pathways. For example, aberrant neddylation has been reported to be involved in several cancers, cardiac and neurodegenerative diseases [25]. Yavuz *et al*. [26] developed a new neddylation site prediction method using a support vector machine algorithm. The feature selection includes protein properties beyond sequence conservation such as hydrophobicity, disorder state and various physicochemical properties. The second PTM prediction method and tool reported in this supplement utilizes maximal dependence decomposition of potential motifs associated with O-linked glycosylation catalyzed by O-GlcNAc transferase [27].

Sowmya and Ranganathan [28] investigated generic features of PPIs that govern binding at the interface of proteins on a quantitative level. Among nine feature classes: interface area, interface polar abundance, interface charged residues percentage, and solvation free energy gain upon interface formation, binding energy turned out to be significantly different. The finding has the potential to improve the characterization of protein complexes at the single-residue level and improve docking methods for predicting protein-protein binding. Large-scale prediction of protein-binding affinities based on sequence properties was addressed by Srinivasulu *et al*. [29] who introduced an SVM with support vector regression feature selection. Fourteen features of physiochemical properties were found to be informative for predicting the affinities of heterodimeric protein complexes. The last paper in this section deals with the issue of antigenic protein surface residues in conformational B-cell epitope prediction. Ren *et al*. [30] developed a weighted SVM algorithm that has been implemented with together with data pre-processing steps as the positive-unlabeled prediction pipeline (PUPre). PUPre performance was evaluated using unbound antigen structures of antigens that circumvents constraints of bound antigen structures. PUPre outperformed existing methods when tested on the three antigens with known epitopes, and it will be interesting to see if the method will be implemented as web tool for use in prediction of unknown B-cell epitopes by a wider user community.

## Bioimaging

Biological image classification is gaining more and more importance beyond cell imaging and diagnostics. Artificial neural networks (ANN) have been successfully applied in the taxonomic classification of algae from images [31]. Here, Kien *et al*. [32] developed an automated ANN-based species identification technique for copepod zooplankton using dorsal microscopy images. The methodology shows promise to reduce the time spent on the taxonomic analysis of plankton in aquatic ecosystem studies. The focus of the second bioimaging paper is on single-cell Raman spectra of bacteria [33]. The authors present an improved method that transforms the Raman spectrum into a discrete spectrum that results into rapid spectra comparison and higher classification accuracy.

## Genomics

Light-RCV [34] is another next-generation sequencing reads and alignment viewer. The genome-wide read coverage at base level is facilitated by a memory dump of the coverage which does not require lengthy sequential loading of a specific base position. Kimura and Koike [35] used the Burrows-Wheeler transform to identify the exact position of break points in genomic rearrangements. The exact position is determined without using split reads by applying discordant pairs and a lossless dictionary of reads. When applied to heterogeneous cancer genome sequence samples previously unknown somatic breakpoints were detected. Genomics is increasingly routinely applied in environmental sciences. For example in soil microbiome studies 16S rDNA sequencing is used to determine taxonomic distribution of bacteria in samples. Chen *et al*. [36] designed a sequencing pipeline with reduced primer bias and post-processing that resulted in longer 16S rDNA sequences during a proof-of-principle sequencing of dioxin-containing soil samples. In microbial metagenomic studies the number of mixed bacterial populations is termed richness. Jayasundara *et al*. [37] improved the estimation of richness for viral strains using quasispecies spectra obtained with a new probabilistic method.

## Privacy

In 2012 the US Presidential Commission for the Study of Bioethical Issues released a report on "privacy and progress in whole genome sequencing" [38]. The analysis results and recommendations range from policies and ethics to technical issues such as computational access. Database developers and providers face the conundrum of promoting data access and sharing data while providing protection, security and privacy. In the past three years a few papers explored fuzzy encryption to deal with privacy issues when searching genotype and single-nucleotide polymorphism (SNP) data [39, 40]. Shimizu *et al*. [41] proposed an additive-homomorphic cryptosystem for protecting user and database privacy. The results of a case-study on searching a large chemical compound database have been encouraging and may result in future applications in drug discovery-related searching of sensitive data and searching for SNPs in personal genome databases.

## Conclusion

The articles in the two GIW/InCoB2015 supplements of BMC Bioinformatics and BMC Systems Biology represent a variety of new bioinformatics methods that will enable new studies to advance our biological and biomedical knowledge on molecular and system levels. The next opportunity to present and publish innovative bioinformatics tools and novel bioinformatics-driven research in the framework of InCoB and collaborating partners is coming soon. InCoB2016 will be held from September 21-23, 2016 in Singapore [42].

## List of abbreviations used

AASBi: - Association of Asian Societies for Bioinformatics
ANN: - artificial neural network
APBioNet: - Asia-Pacific Bioinformatics Network
CNA: - copy number alteration
GIW: - International Conference on Genome Informatics
HMM: - Hidden Markov Model
IEEE/ACM: - IEEE Communications Society/Association for Computing Machinery
InCoB: - International Conference on Bioinformatics
JSBi: - Japanese Society for Bioinformatics
PPI: - protein-protein interaction
PTM: - post-translational modification
SNP: - single-nucleotide polymorphism
TF: - transcription factor.
